# Finger-Vein Verification Based on Multi-Features Fusion

**DOI:** 10.3390/s131115048

**Published:** 2013-11-05

**Authors:** Huafeng Qin, Lan Qin, Lian Xue, Xiping He, Chengbo Yu, Xinyuan Liang

**Affiliations:** 1 School of Computer Science and Information Engineering, Chongqing Technology and Business University, Chongqing 400030, China; E-Mails: jsjhxp@ctbu.edu.cn (X.H.); lxy3400@sina.com (X.L.); 2 Key Laboratory of Optoelectronic Technology and Systems of Ministry of Education, College of Opto-Electronic Engineering, Chongqing University, Chongqing 400030, China; E-Mails: qinlan@cqu.edu.cn (L.Q.); xuelian@cqu.edu.cn (L.X.); 3 College of Electronic and Automation, Chongqing University of Technology, Chongqing 400045, China; E-Mail: yuchengbo@cqut.edu.cn

**Keywords:** personal identification, finger-vein, scale invariant feature transform, orientation encoding, multi-features fusion

## Abstract

This paper presents a new scheme to improve the performance of finger-vein identification systems. Firstly, a vein pattern extraction method to extract the finger-vein shape and orientation features is proposed. Secondly, to accommodate the potential local and global variations at the same time, a region-based matching scheme is investigated by employing the Scale Invariant Feature Transform (SIFT) matching method. Finally, the finger-vein shape, orientation and SIFT features are combined to further enhance the performance. The experimental results on databases of 426 and 170 fingers demonstrate the consistent superiority of the proposed approach.

## 1. Introduction

With the growing demand for more user friendly and stringent security, automatic personal identification has become one of the most critical and challenging tasks. Thus, some researchers are motivated to explore new biometric features and traits. The physical and behavioral characteristics of people, i.e., biometrics, have been widely employed by law enforcement agencies to identify criminals. Compared to traditional identification techniques such as cards, passwords, the biometric techniques based on human physiological traits can ensure higher security and more convenience for the user. Therefore, the biometrics-based automated human identification are now becoming more and more popular in a wide range of civilian applications. Currently, a number of biometric characteristics have been employed to achieve the identification task and can be broadly categorized in two categories: (1) extrinsic biometric features, *i.e.*, faces, fingerprints, palm-prints and iris scans; (2) intrinsic biometric features, *i.e.*, finger-veins, hand-veins and palm-veins. The extrinsic biometric features are easy to spoof because their fake versions can be successfully employed to impersonate the identification. In addition, the advantages of easy accessibility of these extrinsic biometric traits also generate some concerns on privacy and security. On the contrast, the intrinsic biometric features do not remain on the capturing device when the user interacts with the biometrics device, which ensures high security in civilian applications. However, there are limitations in palm-vein and hand vein verification systems due to the larger capture devices required. Fortunately, the size of finger-vein capture devices can be made much smaller so that it can be easily embedded in various application devices. Moreover, using the finger for identification is more convenient for the users. In this context, personal authentication using finger-vein features has received a lot of research interest [1–17].

Currently, many methods are developed to extract vein patterns from the captured images with irregular shading and noise. Miura [5] *et al.* proposed a repeated line tracking algorithm to extract finger-vein patterns. Their experimental results show that their method can improve the performance of the vein identification. Subsequently, to robustly extract the precise details of the depicted veins, they investigated a maximum curvature point method [6]. The robustness in the extraction of finger-vein patterns can be significantly improved based on calculating local maximum curvatures in cross-sectional profiles of a vein image. Zhang [7] *et al.* have successfully investigated finger-vein identification based on curvelets and local interconnection structure neural networks. The Radon transform is introduced to extract vein patterns and the neural network technique is employed for classification in reference [8]. The performance using this approach is better than that of other methods. Lee and Park [9] have recently investigated finger-vein image restoration methods to deal with skin scattering and optical blurring using point spread functions. Experimental results suggest that the performance of finger-vein recognition using restored finger images can be improved significantly. In our previous work [10], an effective method based on minutiae feature matching was proposed for finger-vein recognition. To further improve performance, a region growth-based feature extraction method [11] is employed to extract the vein patterns from unclear images. For a small database, the two methods can achieve high accuracy by matching these images. Currently, a wide line detector is being investigated for finger-vein feature extraction by Huang *et al.* [12]. Their experimental results have shown that a wide line detector combined with pattern normalization can obtain the best results among these methods. Meanwhile, a new finger-vein extraction method using the mean curvature [13] is developed to extract the pattern from the images with unclear veins. As the mean curvature is a function of the location and does not depend on the direction, it achieves better performance than other methods.

The vein feature extraction methods described above have shown better performance for finger-vein recognition, however, they have the following limitations: (1) as some of the pattern extraction methods such as maximum curvature [6] and mean curvatures [13] emphasize the pixel curvature, the noise and irregular shading are easily enhanced. Thus, they cannot detect effective vein patterns for authentication; (2) The methods described above only focus on single feature extraction (the shape of veins), rather than multi-feature extraction. However, it is difficult to extract a robust vein pattern because the captured vein images contain irregular shading and noise, therefore, only by using the shape of vein patterns one cannot achieve robust performance in finger-vein recognition; (3) The matching scores generated from these methods are either global or local, so it is difficult to accommodate the local and global changes at the same time. To solve these problems, a new scheme is proposed herein for finger-vein recognition. The main contributions from this paper can be summarized as follows:

Firstly, this paper proposes a new approach which can extract two different types of finger-vein features and achieves a most promising performance. Unlike the existing approaches based on curvature [6,13], the proposed method emphasizes the difference value of the two curvatures in any two orthogonal tangential directions, so the finger region vein can be distinguished from other regions such as the flat region, the isolated noise and irregular shading. Meanwhile, the finger-vein orientation is also estimated by computing the maximum difference value.

Secondly, we proposed a localized matching method to accommodate the potential local and global variations at same time. The localized vein sub-regions are obtained according to feature points which can be determined by the improved feature points removal scheme in the SIFT framework. Then the matching scores are generated by matching the corresponding partitions in two images.

Thirdly, this paper investigates an approach for finger-vein identification combining SIFT features, shape and orientation of finger-veins. As different kinds of features reflect objects in different aspects, the combination strategy should be more robust and improve performance. The experimental results suggest the superiority of the proposed scheme.

The rest of this paper is organized as follows: Section 2 details our proposed feature extraction method. Section 3 describes the matching approach for the finger-vein verification. In Section 4, we obtain combination scores based on two fusion approaches and the experimental results and discussion are presented in Section 5. Finally, the key conclusions from this paper are summarized in Section 6.

## 2. Finger Image Shape Feature Extraction and Orientation Estimation

**Figure 1.**
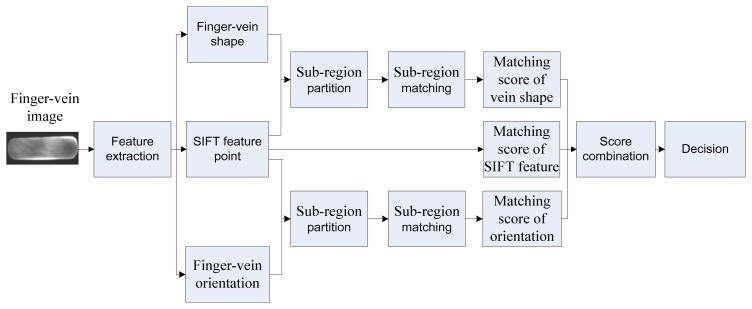
Block diagram for personal identification using finger-vein images.

The block diagram of the proposed system is shown in Figure 1. In this section, we will extract the finger-vein shape and orientation patterns based on the difference curvature.

### 2.1. The Extraction of Finger-Vein Shape Feature

The curvature has been successfully applied in image segmentation, edge detection, and image enhancement. Miura *et al.* [6] and Song *et al.* [13] brought this concept into finger-vein segmentation, and their experimental results have shown that the method based on curvature can achieve impressive performance. However, the two methods based on the curvature only emphasize the curvature of pixel, so the noise and irregular shading in a finger-vein image are easily enhanced. To further extract effective vein patterns, we proposed a new finger-vein extraction method based on curvature of pixel difference, which is shown as follows.

Suppose that *F* is a finger-vein image, and *F*(*x*, *y*) is the gray value of pixel (*x*, *y*). A cross-sectional profile of point (*x*, *y*) in any direction is denoted by *P*(z). Its curvature is computed as follows:

(1) $$K(z)=\frac{\left|P^{\prime \prime }(z)\right|}{{\{1+P^{\prime }{\left(z\right)}^{2}\}}^{3/2}}$$

where *P″*(z)=d^2^*P*/ *dz*^2^ and *P′*(z)=d*P*/ *dz*.

Therefore, the maximum difference curvature can be defined as:

(2) $$D_{\text{max}}=\underset{0\leq \theta \leq \pi }{\text{max}}\Delta K_{\theta }$$

where $\Delta K_{\theta }=\left\{ \begin{array}{ll} \begin{array}{c} K_{\theta }(z)-K_{\theta +\pi /2}(z)\;if\;\theta \leq \pi /2\\ K_{\theta }(z)-K_{\theta -\pi /2}(z)\;if\;\theta >\pi /2 \end{array} & \left(0<\theta \leq \pi \right) \end{array}\right.$, and *K_θ_*(z) and *K*_*θ*+*π*/2_(z) represent the curvatures in the direction *θ* and the direction perpendicular to *θ*, respectively. The enhancement vein image is obtained after computing maximum difference curvature of all pixels. Then the vein pattern is binarized using a threshold. It is worthwhile to highlight several aspects of the proposed method here:

1. For the vein regions, the curvature is large in ridge direction and small in the direction perpendicular to the ridge direction. Therefore, *D_max_* is large.
2. For the flat regions, the curvatures in all directions are small, so the maximum differences *D*_max_ are small.
3. For the isolated noise and irregular shading, the curvature in all directions is large, but *D_max_* is still small.

According to the analysis above, the vein region can be distinguished from other regions effectively, so the difference curvature method can obtain robust vein patterns.

### 2.2. Orientation Estimation

The orientation encoded method is applied to palm-prints [18] and palm-veins [19] and has shown high performance. Therefore, we attempt to preserve the orientation features of finger-vein by the following encoding method:

#### [Step1] Determination of Orientation

**Figure 2.**
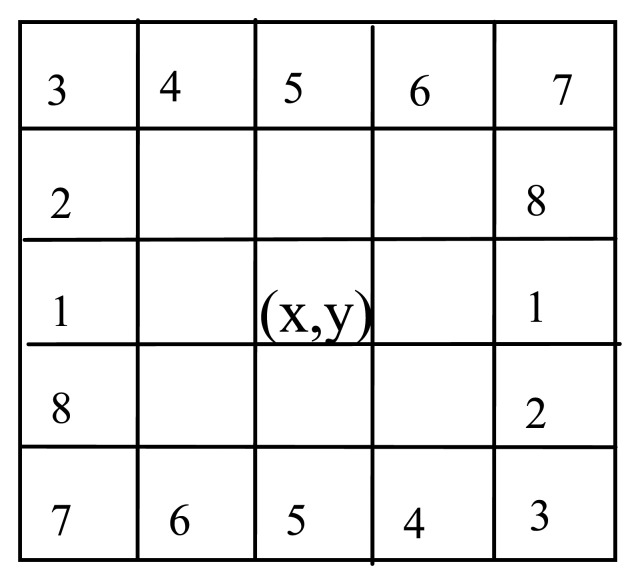
Eight directions of a pixel.

As the finger-vein extends along the finger, the finger-vein has a clear orientation field. To estimate the orientation, we divide the ridge direction of a pixel (*x*, *y*) into eight directions, which is shown in Figure 2. Then the eight directions are divided into four groups and the two directions in each group are perpendicular to each other. Let G*_j_* = {*j*, *j* + 4} be *j*th group.

#### [Step2] Computation of Difference Curvature

The curvatures in two directions *G_j_* can be computed using Equation (1), respectively. The difference values of the curvatures in each group are calculated as:

(3) $$\Delta K_{\text{j}}=\left\{ \begin{array}{ll} \begin{array}{c} K_{j}\left[z\right]-K_{j+4}\left[z\right]\;if\;j\leq 4\\ K_{j}\left[z\right]-K_{j-4}\left[z\right]\;if\;j>4 \end{array} & \left(j=1,2,\ldots ,8\right) \end{array}\right.$$

#### [Step 3] Encoding The Orientation of Vein

Based on Equation (3), the ridge orientation of pixel (*x*, *y*) is determined as follows:

(4) $${\text{j}}_{\max}=\text{arg}\{\underset{\text{j}\in \{1,2,\ldots ,8\}}{\text{max}}(\Delta K_{\text{j}})\}$$

It should be pointed out that the largest rotational changes this orientation encoding scheme can address is *π*/8, since the directions of all pixels are quantized to only eight orientations. If the number of quantized orientations is too small, the encoding scheme is robust to the rotation variations, and not distinguishable. On the contrast, it is sensitive to the rotation variations and the genuine match. Therefore, the performance of the encoding scheme depends on number of orientations. In our work, the number of quantized orientations is set to 8.

## 3. Sub-Region Matching Method

Currently, there are two major vein matching methods: the minutia-based matching method [10,14,15] and the shape-based matching method [5,6,11,13]. These matching methods using minutia can achieve high accuracy for high resolution vein images. Unfortunately, the minutia points are easily disturbed and lost for low resolution vein images, which can significantly reduce the performance. Therefore, most researchers employ the vein shape to match two images, and develop some matching methods [5,6] to address translation variations in both horizontal and vertical directions. However, these matching methods are sensitive to the potential deformation because the matching scores generated from them are the global matching scores. In most cases, the matching scores among the localized vein regions (in two images) are more robust to local/partial distortions. Therefore, some researchers [19] use the sub-region matching method to overcome the local deformation. Unfortunately, as the sub-region matching method partitions an image into different non-overlapping blocks, it can not overcome the global variations such as whole translation or rotation between two images. Therefore, existing vein matching methods [3–14,16,17,19] cannot accommodate the local changes (local deformation) and global variations (global translation, and global rotation) at the same time. To overcome this drawback, we propose a new localized matching method based on the SIFT feature. Firstly, an improved SIFT match method is employed to determine the correct matching points. According to these matching points, a finger image is partitioned into many nonoverlapping or overlapping localized sub-regions. Then the match scores are obtained by matching the corresponding partitions in two images. Finally, we combine the matching scores of each patch.

### 3.1. Determination of Feature Points Based on the Improved Sift Matching Method

SIFT is a very powerful local descriptor, which is invariant to image scale, translation and rotation, and is shown to provide robust matching across a substantial range of affine distortion, addition of noise, and changes in illumination. Recently, the local descriptor was employed for palm-vein recognition [20] and hand vein recognition [21].

SIFT description includes four steps [22]: (1) Scale-space extremum detection; (2) Feature points localization; (3) Orientation assignment; (4) Feature points descriptor. After processing by above steps, each image is described by a set of the 128-elements SIFT invariant features. Let *P*={*p*_i_}, *i* = 1,2…*m* and *Q* = {*q_j_*}, *j* = 1,2…*n* are the feature point sets of gallery and probe image respectively, where *m* and *n* are the number of SIFT feature. The matching method proposed by Lowe [22] is as follows.

The Euclidean distances between a gallery feature point and all the SIFT features in the probe image are computed by:

(5) $$d_{i}(p_{i},q_{i}^{*})=\min_{j=1,2,\ldots ,n}d_{ij}\;(i=1,2\ldots m)$$

where *d_j_* = *d*(*p_i_*,*q_j_*) denotes the Euclidean distance between two SIFT descriptors *p_i_* and *q_j_*. $q_{i}^{*}$ is the closest neighbor point of *p_i_*.

However, many features from a gallery image will not have any correct matches in the probe image because some feature points were not detected in the probe image. To enhance matching performance, these mismatching feature points are discarded by comparing the distance of the closest neighbor to that of the second-closest neighbor:

(6) $$d^{\prime }=d_{i}\;(p_{i},q_{i}^{*})\;if\;d_{i}\;(p_{i},q_{i}^{*})\leq cd_{i}(p_{i},q_{i}^{\prime })$$

where $q_{i}^{\prime }$ is the second-closest neighbor point of *p_i_*, and *c* is a constant.

This method is an effective way to remove mismatching feature points detected from the image where there are sharp changes between different regions of an image. However, finger-vein images are non-rigid, round and smooth objects and contain few straight edges. The intensity changes in finer vein images are gradual and slow, so the blob and corner structures are not significantly different from their neighboring pixels. Therefore, it is difficult to remove mismatching feature points in the finger-vein images using Equation (6), which can degrade the matching accuracy. To solve this problem, we use following method to remove the mismatching points.

Based on Equation (5), we can obtain *m* SIFT distances $d_{i}(p_{i},q_{i}^{*})\;i=1,2,\ldots ,m$. Let (*x_i_*,*y_i_*) and $\left(x_{i}^{*},y_{i}^{*}\right)$ be the spatial positions of a gallery feature point *p_i_* and a probe feature point $q_{i}^{*}$. The geometric distance $g(p_{i},q_{i}^{*})$ between *p_i_* and $q_{i}^{*}$ is computed as:

(7) $$g(p_{i},q_{i}^{*})=\sqrt{{\left(x_{i}-x_{i}^{*}\right)}^{2}+{\left(y_{i}-y_{i}^{*}\right)}^{2}}$$

**Figure 3.**
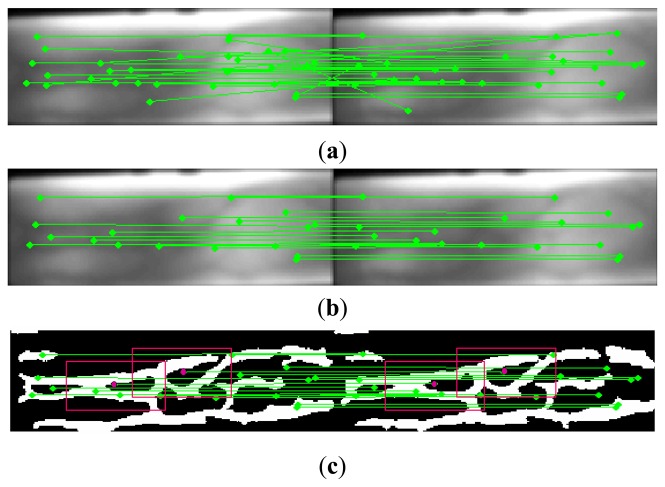
Matching results of SIFT features for finger-vein images from a same person. (**a**) Feature points obtained by original SIFT method; (**b**) Feature points obtained by improved method; (**c**) The sub-regions for two pairs of matching points.

The *m* geometric distances $g(p_{i},q_{i}^{*})$ are arranged in order of increasing number and form a new array ${\left\{g({p^{\prime }}_{i},{q^{\prime }}_{i})\right\}}_{i=1}^{m}$. The feature points corresponding to the *k* smallest geometric distances of ${\left\{g({p^{\prime }}_{i},{q^{\prime }}_{i})\right\}}_{i=1}^{m}$ are denoted by ${\left({p^{\prime }}_{i},{q^{\prime }}_{i}\right)}_{i=1}^{k}$, where the *k* is determined as:

(8) $$k=\left\{ \begin{array}{ll} T & if\;T\leq m\\ m & \text{else} \end{array}\right.$$

where *m* is the total number of the feature points which is usually different for each pair of different images, and *T* is threshold which is set to 20 experimentally. Therefore, *k* pairs of feature points ${\left({p^{\prime }}_{i},{q^{\prime }}_{i}\right)}_{i=1}^{k}$ are remained as the matching points. On the contrast, other *m-k* feature points are discarded as the mismatching points. Figure 3b shows 20 pairs of matching feature points obtained from Figure 3a by our removal scheme.

### 3.2. Generating Sift Score and Sub-Region from Gallery and Probe Images

The *k* pairs of matching feature points are generated from gallery and probe images (original grayscale images) in the previous section. Based on these feature points, the SIFT distance between a gallery and probe image is computed by:

(9) $$\text{sift}=\frac{1}{\text{k}}\sum_{i=1}^{k}d({p^{\prime }}_{i},{q^{\prime }}_{i})$$

In addition, the vein shape or orientation image can be divided into *k* nonoverlapping or overlapping sub-regions using the spatial positions of *k* feature points. Let *A* and *B* represent the template generated from the gallery and probe finger-vein images (vein shape or orientation), respectively. Then the *k* sub-regions from *A* and *B* are denoted by:

(10) $$A^{\prime }=\{{a_{i}}^{w_{a},h_{a}}|{a_{i}}^{w_{a},h_{a}}\;\in \;A,i=1,2\ldots ,k\}$$

(11) $$B^{\prime }=\{{b_{i}}^{w_{b},h_{b}}|{b_{i}}^{w_{b},h_{b}}\;\in \;B,i=1,2\ldots ,k\}$$

where ${a_{i}}^{w_{a},h_{a}}$ and ${b_{i}}^{w_{b},h_{b}}$ denote the localized sub-regions separated from *A* and *B*, *w_a_* and *h_a_* are width and height of sub-region ${a_{i}}^{w_{a},h_{a}}$, and *w*_b_ and *h_b_* are width and height of sub-region ${b_{i}}^{w_{b},h_{b}}$ (*w_a_* > *w_b_*, and *h_a_* > *h_b_*). The center point of each sub-region is the matching feature points. Figure 3c shows the relationship between sub-region and feature point in a finger-vein shape image.

### 3.3. Generating the Matching Scores of Finger-Vein Shape Images

Suppose that the sub-regions *A*′ and *B*′ are generated from the gallery and probe finger-vein shape images *A* and *B*, respectively. The matching scores between *A* and *B* are computed as:

(12) $$S(A,B)=S(A^{\prime },B^{\prime })=\frac{1}{k}\sum_{i=1}^{k}\Psi_{i}$$

where Ψ*_i_* is the matching scores between two corresponding sub-regions, which is computed as follows:

(13) $$\Psi_{i}=\underset{\forall x\in [0,w_{a}-w_{b}],y\in [0,h_{a}-h_{b}]}{\text{min}}(\frac{\overset{w_{b}-1}{\underset{u=0}{\text{∑}}}\overset{h_{b}-1}{\underset{v=0}{\text{∑}}}\Phi ({a_{i}}^{w_{a},h_{a}}(u+x,v+y),{b_{i}}^{w_{b},h_{b}}(u,v))}{w_{b}\times h_{b}})$$

*x* and *y* are the amount of translation in horizontal and vertical directions, respectively. Let *P*_1_ and *P*_2_ be the values of the pixels located in the gallery and probe images, respectively. Φ is defined as follows:

(14) $$\Phi (P_{1},P_{2})=\left\{ \begin{array}{ll} 1 & if\;\left|P_{1}-P_{2}\right|=1\\ 0 & \text{otherwise} \end{array}\right.$$

In this approach, the partition scheme based on SIFT descriptor is invariable to global translation, and global rotation, and the matching in Equation (13) addresses the possible local variations by matching the two sub regions correspondingly with a small amount of shifting. Therefore, the SIFT based matching scheme is expected to accommodate possible local and global variations.

### 3.4. Generating the Matching Scores of Finger-Vein Orientation

In Section 2, the finger-vein orientation feature is extracted by the proposed encoding approach. The partition scheme and localized matching method of the orientation encoded template is similar to these of finger-vein shape template. Therefore, the matching scores between two encoded finger-vein templates can be generated using Equation (12), except for the replacement function Φ with following formulation:

(15) $$\Phi (P_{1},P_{2})=\left\{ \begin{array}{ll} 1 & if\;\left|P_{1}-P_{2}\right|\neq 0\\ 0 & \text{otherwise} \end{array}\right.$$

In our paper, the sizes of localized sub-regions generated from the gallery and probe vein shape templates are 120×60 and 110×50, respectively. The sizes of localized sub-regions for two finger-vein orientation templates are 60×40 and 50×30.

## 4. Generating Fusion Matching Scores

The existing finger-vein recognition methods [3–13,16,17] cannot employ the vein orientation pattern but rather use the vein shape pattern for identification. However, the vein pattern may not be effectively extracted due to the conditions of a sensor, the health conditions of humans, illumination variations and so on. Therefore, a single vein pattern cannot work well for the finger-vein recognition task. To resolve this problem, the combination strategy integrating finger-vein features, finger orientation encoding and SIFT feature is proposed to enhance the performance. According to the stage at which the fusion takes place, fusion is performed at three different processing levels such as feature, score, and decision level [23]. Score level fusion is commonly applied in biometric systems because the matching scores remain sufficient distinguishable information for identification. Therefore, after obtaining the finger-vein shape and orientation encoding scores by Equation (12) and the SIFT matching score based on Equation (9), we combine three matching scores at score level based on two commonly used combination strategies (weighted sum and support vector classification, SVM). To improve performance, these scores are normalized by the z-score normalization scheme [24], which is defined as:

(16) $$z^{\prime }=\frac{z-u}{\sigma }$$

where *z* is the matching scores from finer-vein shape, orientation and SIFT features, and *u* and σ are the arithmetic mean and the standard deviation of *z*. Then the normalized scores *z′* are used as the input of combination strategies.

### 4.1. Weighted Sum Rule-Based Fusion

The weighted combination strategy has been highly successful applied in biometrics [25,26]. This approach can achieve separation of the genuine and imposter scores by searching for the linear combination and is represented as follows. Let $\left\{z_{1}^{\prime },z_{2}^{\prime },\ldots ,z_{n}^{\prime }\right\}$ be normalized scores from a finger, where *n* is the number of classifier:

(17) $$zf=\sum_{i=1}^{n}z_{i}^{\prime }\times w_{i}$$

where $\overset{n}{\underset{i=1}{\text{∑}}}w_{i}=1$ and *zf* is the combined matching score. $z_{i}^{\prime }$ denotes the score from the *i* th classifier and *w_i_* represents corresponding weight. In our experiments, the optimal weights for the matching scores were empirically determined.

### 4.2. Support Vector Machines (Svm)-Based Fusion

Currently, SVM has been applied to the classification task in multimodal biometric authentication, such as in [27], and [28] and has shown promising performance. This approach can separate the training data into two classes with a hyperplane that maximizes the margin between them [29,30]. In our experiments, the optimal decision hyperplanes of the SVMs were determined by a radial basis function (RBF) kernel. Let $(z_{j}^{\prime }\;c_{j})\;j=1,2\ldots ,m$ be the training data, where $z_{j}^{\prime }=(z_{1j}^{\prime },z_{2j}^{\prime },\ldots ,z_{nj}^{\prime })$ is a score vector with *n* classifiers and *c_j_* is the corresponding class label. *c_j_* is set to 1 for a genuine score vector sample and −1 for an imposter score vector. After performing the training, a weighted matrix is preserved for classification. At the testing phase, a testing score vector *z* can be classified as a genuine class or an impostor class. The SVM training was achieved with C-support vector classification (C-SVC) in the SVM tool developed by Chang and Lin [31].

## 5. Experimental Results

### 5.1. Database

**Figure 4.**
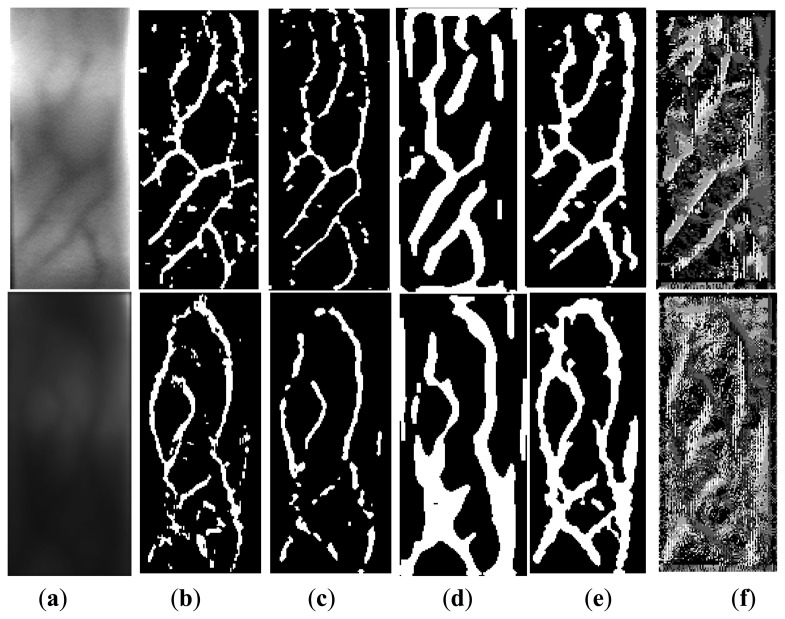
Sample results from different feature extraction methods: (**a**) Finger-vein images from two databases (Top left image from database A and bottom left image from database B); (**b**) Vein pattern from maximum curvature; (**c**) Vein pattern from mean curvature; (**d**) Even Gabor with Morphological; (**e**) Vein pattern from difference curvature; and (**f**) Orientation pattern from difference curvature.

In order to test the performance of the proposed schemes, we performed rigorous experiments on our database and another finger-vein database provided by the National Taiwan University of Science and Technology [32]. All images in the two databases are filtered prior to identification experiments using a two dimensional Gaussian filter with size 5 × 5 pixels and standard deviation 3 pixels. As the distance between the finger and the camera is fixed, the captured images in each database have the same size. Then the proposed method and some previous methods [6,13,33] are employed to enhance the vein images. For fair comparison, the enhancement vein image is binarized using a global threshold. We implement these approaches using MATLAB 7.9 on a desktop with 2 GB of RAM and Intel Core i5-2410M CPU. Figure 4 illustrates the output of various methods.

#### Database A

For our database, all the images were taken against a dark homogeneous background and subject to variations such as translations, rotations and local/partial distortions. This database comprises 4,260 different images of 71 distinct volunteers. Each volunteer provided three finger images (index finger, middle finger and ring finger) from the left and right hands respectively, and each finger has 10 different image samples. Therefore, each volunteer provided 60 images with a size of 352×288 The black background is removed by cropping the original images and the size of the remaining image is 221×83 pixels.

#### Database B

The finger-vein database was built at the Information Security and Parallel Processing Laboratory National Taiwan University of Science and Technology and consists of two parts: Images and Matching Scores. The ‘Matching Scores’ folder consists of 2 × 510 genuine scores and 2 × 57,120 impostor scores generated from left and right-index fingers, respectively. The ‘Images’ folder contains 680 grayscale images of 85 individuals, each subject having four different images from left and right-index finger respectively. Therefore, there are eight image samples for each person. The size of each image is 320×240 pixels. These original images contain a black background, which should degrade the matching accuracy. Thus we crop them to a dimension of 201×90.

### 5.2. Experimental Results on Database A

**Figure 5.**
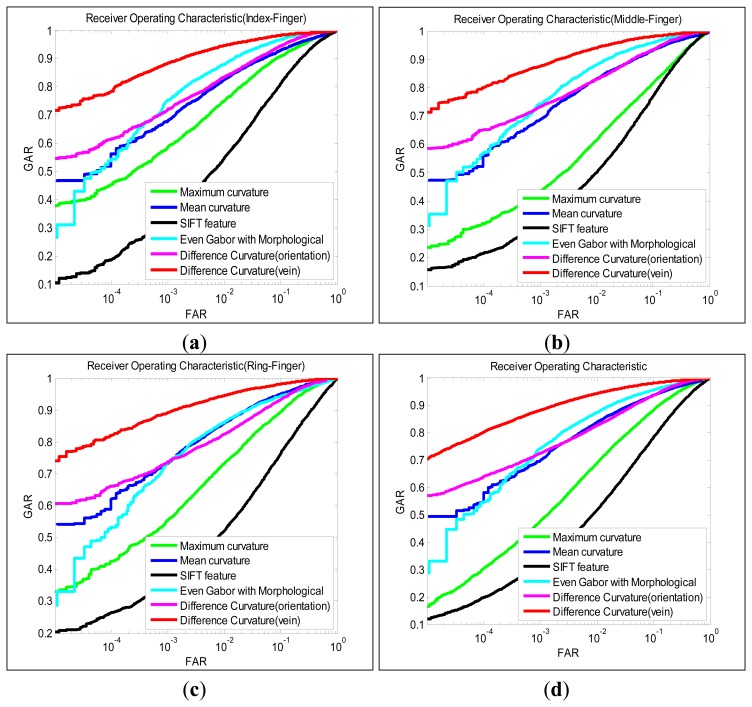
Receiver operating characteristics from finger-vein images (Database A). (**a**) Index-finger (**b**) Middle-finger (**c**) Ring-finger (**d**) Index-finger, middle-finger and ring-finger images.

The objective in this experiment is to evaluate the robustness of the proposed method for a relatively larger finger-vein dataset. Firstly, matching is performed on the index, middle and ring finger images individually, and the matching sets are defined as follows: (1) genuine scores set: matching the ten samples from same fingers to each other, resulting in 6,390 (142 × 45) genuine scores; (2) imposter scores set: the ten finger-vein samples from same finger are randomly partitioned into two subsamples (five for training and the remain for testing). Thus 25 (5 × 5) s 250,275 (142 × 141 × 25/2). Secondly, the different finger images (index, middle and ring finger images) from the same subjects were treated as different classes, thus the total number of classes is 426. Similar to above steps, there are 19,170 (426 × 45) genuine scores and 2,263,125 (426 × 425 × 25/2) imposter scores. Finally, the performance of various feature extraction approaches is evaluated by the equal error rate (EER). The EER is the error rate when the false acceptance rate (FAR) and false rejection rate (FRR) values are equal. The FAR value is the error rate of falsely accepting impostor and the FRR value is the error rate of falsely rejecting genuine. The receiver operating characteristic (ROC) can be obtained by combined FAR and genuine accept rate (GAR) (GAR = 1 – FRR). We compared the performance of our method with the other methods such as SIFT, maximum curvature, mean curvature and even Gabor with morphological. Table 1 lists the EER of the various methods, and the ROC for the corresponding performances is illustrated in Figure 5.

It can be ascertained from Table 1 and Figure 5 that using the difference curvature method to extract vein pattern achieves the best performance among all the approaches referenced in this work. The success results are contributed by emphasizing on the differences among curvatures instead of the magnitude. Therefore, the isolated noise and irregular shading can be removed from the extracted images. The maximum curvature approach and mean curvature approach do not achieve robust performance in our vein database. The poor performance (Table 1 and Figure 5) can be attributed to this fact that the two methods emphasize the curvatures of pixels, so the noise and irregular shading are also emphasized. Therefore, the extraction vein image contains many noises (as shown in Figure 4), which can degrade the performance of finger-vein recognition system. Even Gabor with morphological emphasizes on the shape/structure features of the vein. After the vein patterns are processed by the morphological approach, all the vein lines/curves are processed to similar width. However, the width of the vein lines is actually different from the palm to the finger tip. Therefore, the performance of even Gabor with morphological approach is limited for vein pattern extraction.

In our experiment, the SIFT feature dose not work well on our finger-vein system. This can be explained that the finger-vein image contains less local feature points because it is a non-rigid, round and smooth object. In addition, the performance achieved by the vein orientation is similar to that of the mean curvature method [13], which implies that the orientation of veins also contains important discriminating power. From the experimental results, some performances for the ring finger are better than those for index and middle finger, but it does not imply than the ring finger contains more vein patterns. There is no evidence to show that the performance for one finger should be better than that of another. The different performance for three fingers may be caused by the behavior of the users.

### 5.3. Experimental Results on Database B

**Figure 6.**
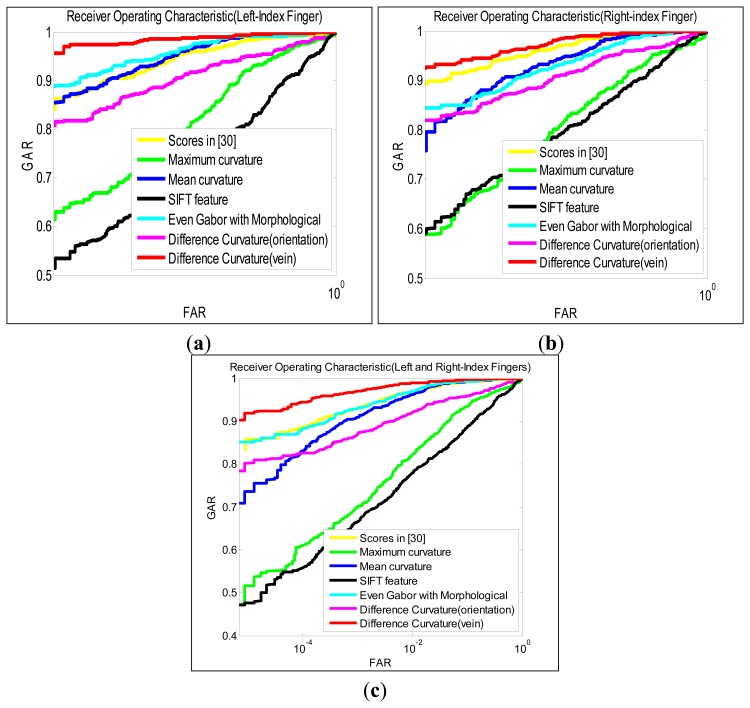
Receiver operating characteristics from finger-vein images (Database B). (**a**) Left index-finger (**b**) Right index-fingers (**c**) Left and right index-fingers.

To further ascertain the robustness of our method, the experimental results on the database B are reported in this section. The performance was firstly evaluated on the left and right-index fingers, respectively. Therefore, the number of genuine score and imposter scores are 510 (85 × 6) and 57,120 (85 × 84 × 16/2) respectively. Similar to experiments A, there are in total 170 classes when different fingers from the same persons that were regarded as belonging to different classes. One thousand twenty (170 × 6) genuine scores and 229,840 (170 × 169 × 16/2) imposter scores are thus generated from the same fingers and different fingers. In addition, reference [32] has also shown the genuine scores and imposter scores generated from their database (referred to Section 5.1 database B), which is computed by the state of art method [3], so the EER and ROC of their approach can be directly obtained based on these matching scores. Therefore, we compare not only these approaches in previous experiments but also the approach in [32] with the proposed method in this experiment. The EER of various methods have been summarized in Table 2. Figure 6 has illustrated the ROC for the corresponding performances.

The experimental results summarized in Table 2 (and Figure 6) are quite similar to the trends from experiments in previous section (experiments A). The proposed method achieves the lowest EER for left-index finger, right-index finger and in combination, respectively. Figure 6 shows that the proposed method can achieve more than 90% GAR at the lower FAR, which is higher than that of other methods.

The experimental results on the databases A and B consistently show that the proposed vein extraction method outperforms other methods in the verification scenario. However, the experimental results on database B in this section are comparatively better than those in previous section generated from database A. This can be explained by the resulting smaller within-class variations, which could be possibly attributed to less finger-vein images from same finger (*i.e.*, only four different images from a finger) in database B.

### 5.4. Performance of Sub-Region Matching Method

**Figure 7.**
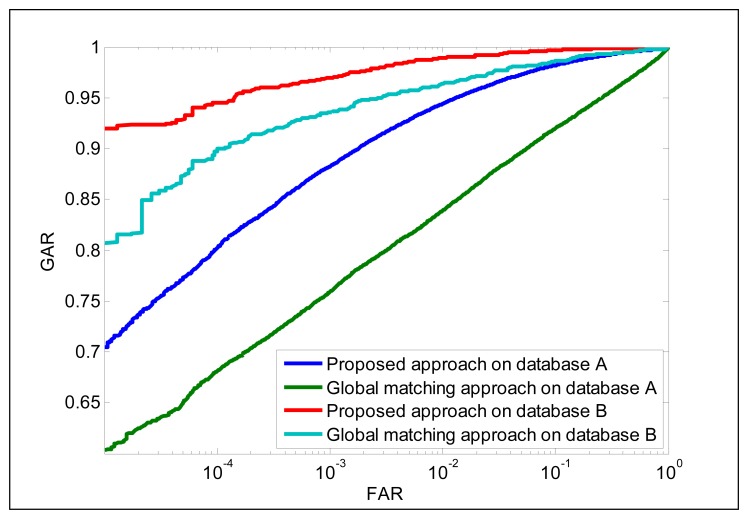
Receiver operating characteristics from two databases for finger-vein shape images with different matching approaches.

The experimental results presented in this section are to estimate the performance of the SIFT-based sub-region matching method. For our approach, the gallery and probe finger-vein images are partitioned into different sub-regions based on feature points and then the matching scores between two corresponding sub-regions are computed using Equation (13). Based on the matching score of each sub-region, the matching score between gallery and probe images can be computed by Equation (12). For the global matching approach, the gallery and probe finger-vein image are used as a whole and submitted into Equation (13), and then the global matching score is obtained. We compare two matching schemes based on finger-vein shape images from two databases (index, middle and ring-finger for database A, and Left and right-index finger for database B). The performance from the two databases using two different matching schemes is shown in Figure 7. It can be observed that the proposed sub-region matching approach has achieved better performance than global matching method. This superior performance can be attributed to the fact that the sub-region matching scheme is more robustness to the local and global variations. In addition, the proposed approach partitions the templates into different sub-regions and thus has increased the training samples to some extent as compared to global matching scheme.

### 5.5. Performance from SIFT Feature, Finger-Vein Shape and Orientation Encoded Combination

In this section, the experimental results are presented to test the performance improvement that can be achieved by combining SIFT feature, finger-vein shape and orientation features based on weighted SUM and SVM fusion rules. For each experiment, half of imposter and genuine scores are randomly selected for training and remaining scores are used for testing. This partitioning of the scores was repeated 20 times, and then we compute the mean of GAR (at certain FAR) and EER on the 20 testing sets to evaluate the performance of finger-vein recognition system. These parameters of z-score normalization were estimated based on training data and three kinds of normalized scores are used as the input of two fusion rules. For the weighted SUM fusion rule, the weights are selected experimentally. For the SVM-based fusion rule, the highest classification accuracy among various kernels such as dot, neural, radial, polynomial and analysis of variance kernels was obtained by a radial-based kernel. The parameters g (gamma in the RBF kernel function) and c (cost of C-SVC function [31]) are set to 0.006 and 1.2 experimentally. The weights of SUM fusion rule and SVM are listed in Table 3.

The experimental results on the database A and database B are summarized in Tables 4 and 5, respectively. For database A, the combined performance achieved in Table 4 is better than those in Table 1. Comparing the results in Tables 2 and 5, there are consistent trends. The experimental results on two databases suggest that the combination of simultaneously acquired finger-vein, finger orientation and SIFT feature can improve the performance. The combined performance on database B is higher than those on database A, which is attributed to the better performance in Table 2 over Table 1 (as discussed in previous section).

## 6. Conclusions

In this paper, we have investigated a novel finger-vein identification scheme utilizing the SIFT, finger-vein shape and orientation features. Firstly, two feature extraction approaches are proposed to obtain finger-vein shape and orientation features. Then we proposed a sub-region matching method to overcome the local and global changes between two vein images. Finally, a combination scheme is employed to improve the performance of finger-vein recognition system. Rigorous experimental results on two different databases have shown that the proposed vein extraction method outperforms previous approaches and a significant improvement in the performance can be achieved by combining SIFT features, finger-vein shape and orientation features.

## Figures and Tables

**Table 1.** Performance from finger-vein matching with various approaches (Database A).

**Approaches**	**Index** **Finger (%)**	**Middle Finger** **(%)**	**Ring Finger** **(%)**	**Index, Middle and** **Ring Finger (%)**
Maximum curvature	9.43	15.4	10.34	10.9
Mean curvature	7.92	8.01	6.35	7.44
SIFT feature	14.5	16.48	17.39	16.09
Even Gabor with Morphological	6.78	5.45	5.15	5.79
Difference curvature d (orientation)	7.08	7.69	7.78	7.59
Difference curvature (vein)	3.34	3.36	3.26	3.32

**Table 2.** Performance from finger-vein matching with various approaches (Database B).

**Approaches**	**Left-Index** **Finger (%)**	**Right-Index Finger (%)**	**Left and Right-Index** **Finger (%)**
Scores in [32]	2.55	1.57	2.16
Maximum curvature	6.68	7.84	7.36
Mean curvature	1.76	2.16	2.06
SIFT feature	11.88	9.61	10.98
Even Gabor with Morphological	1.56	3.14	1.76
Difference curvature (orientation)	4.90	4.61	4.71
Difference curvature (vein)	0.98	1.10	1.08

**Table 3.** Weights of SUM fusion rule and SVM for two databases.

**Fusion Method**	**Database**		**Weights**		

			**SIFT** **Feature**	**Vein** **Shape**	**Orientation** **Encoded**
weighted SUM fusion rule	Database A	Index-finger	0.15	0.7	0.15
		Middle-finger	0.1	0.65	0.25
		Ring-finger	0.15	0.7	0.15
		Index, Middle and Ring-finger	0.1	0.7	0.2

	Database B	Left-index finger	0.1	0.7	0.2
		Right-index finger	0.15	0.75	0.1
		Left and right-index finger	0.1	0.8	0.1

SVM fusion rule	Database A	Index-finger	16.5255	85.3080	42.1578
		Middle-finger	16.7781	78.3649	41.8224
		Ring-finger	15.3110	85.5667	45.9337
		Index, Middle and Ring-finger	48.1544	98.8196	55.8290

	Database B	Left-index finger	6.0424	52.2029	17.9522
		Right-index finger	15.9473	33.6064	24.1848
		Left and right-index finger	12.5155	55.8496	15.4112

**Table 4.** Performance from the combination of SIFT, shape, and orientation of vein (database A).

**Fusion Method**	**Data**	**GAR(%)**	**FAR(%)**	**EER(%)**
weighted SUM fusion rule	Index-finger	86.42	0.0099	2.70
	Middle-finger	86.16	0.0104	2.77
	Ring-finger	86.48	0.0103	2.74
	Index, Middle and Ring-finger	86.56	0.0103	2.74

SVM fusion rule	Index-finger	86.53	0.0104	2.63
	Middle-finger	86.20	0.0103	2.70
	Ring-finger	87.15	0.0103	2.67
	Index, Middle and Ring-finger	86.67	0.0099	2.68

**Table 5.** Performance from the combination of SIFT, shape, and orientation of vein (database B).

**Fusion Method**	**Data**	**GAR(%)**	**FAR(%)**	**EER(%)**
weighted SUM fusion rule	Left-index finger	94.45	0.0101	0.96
	Right-index finger	93.73	0.0105	0.97
	Left and right-index finger	95.01	0.0096	0.79

SVM fusion rule	Left-index finger	94.63	0.0105	0.96
	Right-index finger	94.08	0.0101	0.89
	Left and right-index finger	95.04	0.0096	0.78
